# Cefotaxime Mediated Synthesis of Gold Nanoparticles: Characterization and Antibacterial Activity

**DOI:** 10.3390/polym14040771

**Published:** 2022-02-16

**Authors:** Turki Al Hagbani, Syed Mohd Danish Rizvi, Talib Hussain, Khalid Mehmood, Zeeshan Rafi, Afrasim Moin, Amr Selim Abu Lila, Farhan Alshammari, El-Sayed Khafagy, Mohamed Rahamathulla, Marwa H. Abdallah

**Affiliations:** 1 Department of Pharmaceutics, College of Pharmacy, University of Ha’il, Ha’il 81442, Saudi Arabia; t.alhagbani@uoh.edu.sa (T.A.H.); syeddanishpharmacy@gmail.com (S.M.D.R.); a.abulila@uoh.edu.sa (A.S.A.L.); frh.alshammari@uoh.edu.sa (F.A.); mh.abdallah@uoh.edu.sa (M.H.A.); 2 Department of Pharmacology and Toxicology, College of Pharmacy, University of Ha’il, Ha’il 81442, Saudi Arabia; mdth_ah@yahoo.com; 3 Department of Pharmacy, Abbottabad University of Science and Technology, Havelian 22500, Pakistan; adckhalid@gmail.com; 4 Nanomedicine and Nanotechnology Lab-6 (IIRC), Department of Biosciences, Integral University Lucknow, Lucknow 226026, India; zeddqazi@gmail.com; 5 Department of Pharmaceutics and Industrial Pharmacy, Faculty of Pharmacy, Zagazig University, Zagazig 44519, Egypt; 6 Department of Pharmaceutics, College of Pharmacy, Prince Sattam Bin Abdulaziz University, Al-kharj 11942, Saudi Arabia; e.khafagy@psau.edu.sa; 7 Department of Pharmaceutics and Industrial Pharmacy, Faculty of Pharmacy, Suez Canal University, Ismailia 41522, Egypt; 8 Department of Pharmaceutics, College of Pharmacy, King Khalid University (KKU), Abha 61421, Saudi Arabia; rahapharm@gmail.com

**Keywords:** bacterial resistance, cefotaxime sodium, cephalosporin, gold nanoparticles, MIC_50_

## Abstract

Cefotaxime (CTX) is a third-generation cephalosporin antibiotic with broad-spectrum activity against Gram negative and Gram positive bacteria. However, like other third-generation cephalosporin antibiotics, its efficacy is declining due to the increased prevalence of multidrug-resistant (MDR) pathogens. Recent advances in nanotechnology have been projected as a practical approach to combat MDR microorganisms. Therefore, in the current study, gold nanoparticles (AuNPs) were prepared using cefotaxime sodium, which acted as a reducing and capping agent, besides having well-established antibacterial activity. The synthesized cefotaxime-loaded gold nanoparticles (C-AuNPs) were characterized by UV-Visible spectroscopy, FTIR, TEM and DLS. In addition, the in vitro antibacterial activity of C-AuNPs was assessed against both Gram-positive and Gram-negative bacteria. UV-Visible spectroscopy verified the formation of C-AuNPs, while TEM and DLS verified their nano-size. In addition, CTX loading onto AuNPs was confirmed by FTIR. Furthermore, the colloidal stability of the synthesized C-AuNPs was ascribed to the higher net negative surface charge of C-AuNPs. Most importantly, the synthesized C-AuNPs showed superior antibacterial activity and lower minimum inhibitory concentration (MIC) values against Gram-negative (*Escherichia coli*, *Klebsiella oxytoca*, *Pseudomonas aeruginosa*) and gram-positive (*Staphylococcus aureus*) bacteria, compared with pure CTX. Collectively, CTX was successfully adopted, as reducing and capping agent, to synthesize stable, nano-sized spherical C-AuNPs. Furthermore, loading CTX onto AuNPs could efficiently restore and/or boost the antibacterial activity of CTX against resistant Gram-negative and Gram-positive bacteria.

## 1. Introduction

Cefotaxime sodium (CTX), a cephalosporin sodium salt having [2-(2-amino-1,3-thiazol-4-yl)-2-(methoxyimino)acetyl]amino and acetoxymethyl side groups, is a powerful -lactamase third-generation antibiotic. CTX has broad-spectrum activity against both Gram-positive and Gram-negative bacteria, and it shows higher activity than first and second-generation cephalosporins, especially against Gram-negative bacteria [1]. CTX is known to exert its antibacterial activity by attaching to penicillin-binding proteins (PBPs) with its beta-lactam rings, blocking transpeptidation step in peptidoglycan, a key protective barrier of the bacterial cell wall, formation in susceptible microorganisms, and thus, resulting in autolysis [2].

Despite the fact that several antibiotics are available commercially for the treatment of both Gram-positive and Gram-negative bacteria, their extensive use has led in developing resistance in these pathogens; antibiotic resistance constitutes a significant challenge in global public health [3]. For instant, bacteria have reportedly acquired a novel form of β-lactamase enzyme that results in conferring resistance to antibiotics containing β-lactam ring such as cefotaxime [4,5,6]. Changing the structure of antibiotics’ β-lactam moiety [7,8] and synthesizing new antibiotics with higher potencies [9] are two important strategies used for overcoming bacterial resistance. Nevertheless, these approaches are time consuming and are frequently not economically viable [10].

Instead, nano-strategies for combating multidrug resistant bacteria have emerged as a viable means to overcome the aforementioned problems associated with bacterial resistance [11,12,13]. Recent advances in nanotechnology provide new opportunities for the development of innovative delivery vehicles based on unique types of nanoparticulate systems with varying sizes and shapes and malleable antibacterial activities. One of the most appealing features of nanoparticle-based formulations is their capacity to deliver a wide arsenal of drugs, either attached to their surfaces or entrapped within their core structure, to the site of infection efficiently and safely [14,15,16]. By enhancing the pharmacokinetic behavior and, thereby, pharmacological activity of entrapped/loaded drug entities, compared to free drug counterparts, the administered dose needed to produce the designated therapeutic effect can be reduced considerably [15,17]. As a result, the toxicity and undesirable side effects encountered upon the frequent dosing and/or elevated drug’s plasma concentration can be reduced.

While various materials, ranging from liposomal [18,19] to polymer-based nano-drug drug delivery systems [17,20], have been investigated, metallic nanoparticles, such as gold nanoparticles, are appealing as core materials owing to their inert and nontoxic nature [21,22]. Gold nanoparticles are considered attractive delivery vehicles that can enhance the antibacterial activities of loaded antimicrobial agents [23,24]. Gold nanoparticles (AuNPs), when modified and/or coupled with other antibacterial agents, can display a superior antibacterial activity that might help in combating resistance bacteria [21,25,26]. Many reports have emphasized the efficacy of gold nanoparticles to sensitize resistant bacterial strains to the antibacterial effects of loaded antibiotics [26,27]. For instance, the antimicrobial efficacy of ampicillin was restored against multiple-antibiotic-resistant strains of *E. aerogenes* and *Ps. aeruginosa* upon loading onto gold nanoparticles [28]. Similarly, Rai et al. [10] have demonstrated that cefaclor-loaded gold nanoparticles showed potent antibacterial activity against both Gram-negative (*E.scherichia coli*) and Gram-positive (*S. aureus*) bacteria, compared to either free cefaclor or gold nanoparticles alone. Alshammari et al. [26] have also recently reported that ceftriaxone mediated synthesized gold nanoparticles exerted a two-fold increase in the antibacterial activity against *S. abony, S. aureus, E. Coli*, and *K. pneumonia,* compared to pure ceftriaxone. Interestingly, the photothermal-based bacterial eradicating activity of gold nanoparticles, exploiting the surface plasmon resonance of AuNPs, was demonstrated in several studies, either independently or in conjugation with drugs [29,30,31]. Collectively, these findings emphasized the potential of gold nanoparticles as efficient carriers for antibiotics targeting drug-resistance bacteria.

In this study, therefore, we aimed at challenging the efficiency of gold nanoparticles in boosting the antimicrobial activity of the β-lactamase antibiotic cefotaxime (CTX). For this purpose, we introduced a novel one one-pot synthesis method for the preparation of cefotaxime-loaded gold nanoparticles (C-AuNPs), in which CTX itself was utilized as a reducing and capping agent to convert gold salts into AuNPs, instead of using an external reducing/capping agent. In addition, CTX was loaded simultaneously on the surface of the synthesized AuNPs without the need for conjugating agent. The synthesized CTX-loaded AuNPs (C-AuNPs) were characterized by a UV-Visible spectroscopy, dynamic light-scattering technique, transmission electron microscopy, and Fourier transmission infrared spectroscopy. Finally, the antibacterial activity of the synthesized nanoparticles were challenged against various strains of bacteria.

## 2. Materials and Methods

### 2.1. Materials

All the solvents, antibiotic and synthetic compounds used in the study were of analytical grade and were purchased from Sigma Aldrich (St. Louis, MO, USA). Mueller-Hinton agar was provided by Hi-media (Mumbai, India)

### 2.2. Bacterial Strains and Growth Conditions

Gram-negative strains; *Escherichia coli* (ATCC 25922), *Klebsiella oxytoca* (ATCC 13182) and *Pseudomonas aeruginosa* (ATCC 15692) and Gram-positive *Staphylococcus aureus* (ATCC 25923) were adopted to assess the antibacterial activity of C-AuNPs. Fresh inoculum for each bacterial strain was prepared in Luria–Bertani (LB) broth and incubated at 37 °C for 20 h. Before antibacterial activity, the turbidity of the culture was adjusted to the 0.5 McFarland standard, equivalent to 1.5 × 10^8^ CFU/mL, using LB broth.

### 2.3. Synthesis of Gold Nanoparticles

Gold nanoparticles (AuNPs) were synthesized by incubating 1 mM aqueous gold salt solution (H[AuCl_4_]) in phosphate buffer (pH = 7.4) and CTX (50 µg/mL) in a 3 mL reaction mixture. The reaction mixture was then incubated at 40 °C for 48 h. As a control, a reaction mixture containing just antibiotic (CTX) was utilized. Following a 48-h incubation period, the solution color changes to ruby red, demonstrating that the reaction has been completed. The generated cefotaxime-loaded gold nanoparticles (C-AuNPs) were then recovered by centrifugation (30,000× *g*) for 30 min. To remove unbound CTX, C-AuNPs were rinsed using 50% *v/v* ethanol and washed two times with Milli Q water.

### 2.4. Characterization of Synthesized C-Gold Nanoparticles (AuNPs)

#### 2.4.1. Ultraviolet (UV)–Visible Spectroscopy

The Shimadzu UV-1601 dual-beam spectrophotometer (Tokyo, Japan) was used to investigate the transformation of gold salts into gold nanoparticles. The UV–Visible spectra of the synthesized C-AuNPs at a resolution of 1 nm within the range of 200–800 nm was recorded. This method relies on the phenomenon that reducing gold salts to synthesized AuNPs led to a color change.

#### 2.4.2. Transmission Electron Microscopy (TEM)

TEM was employed to determine the size and shape of C-AuNPs. Briefly, one drop of AuNPs suspension was put onto a carbon-coated TEM copper grid and allowed to air dry. Any remaining solution was removed with filter paper. C-AuNPS were then examined with a Tecnai G2 Spirit TEM outfitted with a BioTwin lens configuration (Hillsboro, OR, USA). The set-up is powered by an accelerating voltage of 80 kV.

#### 2.4.3. Dynamic Light Scattering (DLS) Analysis

The hydrodynamic diameter of the produced nanoparticles was determined via dynamic light-scattering (DLS) technique using Malvern Zetasizer Nano-ZS (ZEN3600, Malvern Instrument Ltd., Malvern, UK). The sample was taken in a 1.5 mL DTS0112 disposable low-volume cuvette. Before measuring, the sample was sonicated for 1 min and filtered using syringe membrane filters with pore size <0.45 µm. The mean particle size was calculated by taking the average of three measurements of a single sample.

For the measurement of zeta potential (particle surface charge), a Malvern Zetasizer Nano-ZS (Malvern Instrument Ltd., Malvern, UK) was adopted. Samples were prepared in a similar way as for the DLS measurement, and DTS1070 disposable cuvettes were used for zeta potential analysis.

#### 2.4.4. Fourier Transform Infrared (FTIR) Spectroscopy

FTIR spectroscopy was employed to confirm the attachment/loading of CTX onto AuNPs. For FTIR analysis, a film was prepared by placing a drop of C-AuNPs solution on Si(111) substrate, and excess water droplets were removed by delicate warming. FTIR spectra were then recorded using a Shimadzu FTIR-8201 PC apparatus (Tokyo, Japan). To get the significant signal to noise ratio, 256 outputs of the prepared film were recorded in the range of 400–4000 cm^−1^.

### 2.5. Determination of Loading Efficiency of Cefotaxime (CTX) onto C-AuNPs

To estimate CTX loading efficiency onto C-AuNPs, the produced C-AuNPs were recovered from the reaction mixture by centrifugation for 30 min at 30,000× *g*, and the supernatant was separated. The concentration of free cefotaxime in the supernatant was determined spectrophotometrically at λ_max_ of 260 nm [32], using a pre-established calibration curve of CTX at various concentrations. The percentage loading efficiency was determined using the following equation:

$$\mathrm{Loading}\;\mathrm{efficiency}\;(\%)=\left(\left(A-B\right)/A\right)\times 100$$

where, *A* is the total amount of CTX added during C-AuNPs synthesis, while *B* is the amount of free CTX in the supernatant of C-AuNPs.

### 2.6. Assessment of Antibacterial Activity

#### 2.6.1. Agar Well Diffusion Method

The antimicrobial action of pure CTX and C-AuNPs was determined using the agar well diffusion method. A 100 µL fraction of each microbial inoculum (namely, *Eschrichia coli*, *Klebsiella oxytoca*, *Pseudomonas aeruginosa* and *Staphylococcus aureus*) was obtained using a micropipette to ensure a uniform lawn of cells onto the agar plates. The agar plates were inoculated by evenly swabbing across the whole surface of the plate three times and rotating the Petri plates at a 60° angle after each application. Following that, a hole (6 mm in diameter) was aseptically punched with a sterile cork tip, and 100 µL of C-AuNPs (4.19 µg/well; as determined by loading efficiency) and pure CTX (20 µg/well) were poured into the wells, however, phosphate buffer saline (PBS) was used as a control. The agar plates were then incubated under appropriate conditions overnight at 37 °C. Post-incubation, the Petri plates were examined for the zone of inhibition, which was quantified in millimetres using a millimetre scale. To prevent the error, the experiments were performed three times. The inhibitory zone was determined as mean ± standard deviation.

#### 2.6.2. Determination of Minimal Inhibitory Concentration (MIC)

The broth microdilution method was adopted to estimate the MICs of synthesized C-AuNPs against different bacterial strains, as described previously [33]. Briefly, in a 96-well plate, aliquots of 10 μL of each bacterial inoculum (1 × 10^5^ CFU/mL) were inoculated to each well. Subsequently, serial dilutions of the synthesized C-AuNPs, in sterilized double distilled water, within the concentration range of 0.46 μg/mL were then added to each well. Additionally, aliquots of free cefotaxime (0.46 to 30 μg/mL) were added to each well for comparison. After adding the CTX and C-AuNPs, the plates were further incubated at 37 °C for 24 h. Afterwards, cell viability was assessed using an ELISA plate reader at 625 nm. The MIC was the lowest concentration of synthesized C-AuNPs or free CTX that efficiently suppressed the bacterial growth after overnight incubation. PBS was added as a negative control, and the results obtained represented the mean ± SD of three independent experiments

## 3. Results and Discussion

### 3.1. Synthesis of Cefotaxime-Loaded Gold Nanoparticles (C-AuNPs)

**Figure 1:**
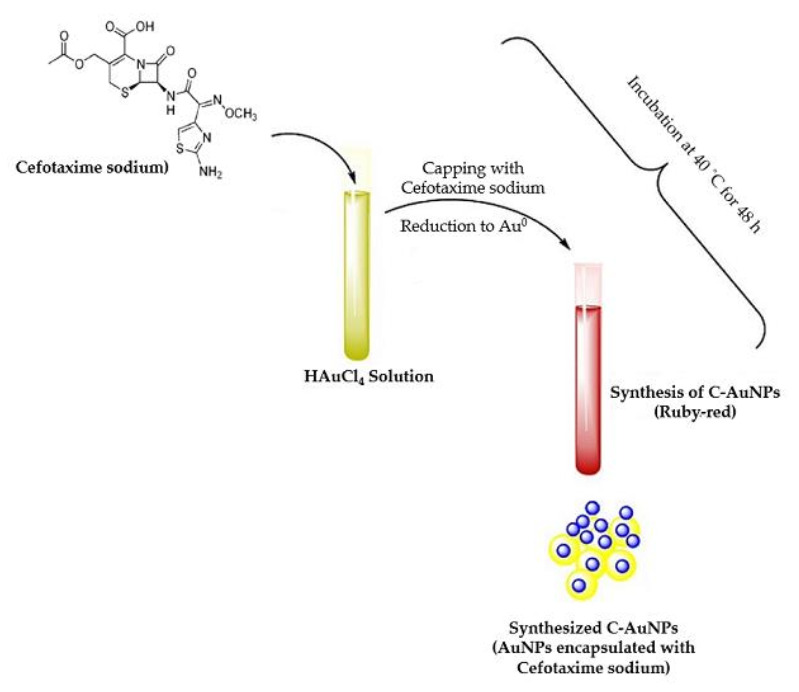
Diagrammatic representation of C-gold nanoparticles (AuNPs) synthesis procedure.

Gold nanoparticles (AuNPs) have been widely utilized in the field of bionanotechnology because of their unique characteristics and diverse surface activities [34,35]. Several techniques have been adopted for the synthesis of AuNPs [36,37]. All these techniques rely principally on chemicals or biomolecules that can serve as reducing and capping agents to convert gold salts into gold nanoparticles. Furthermore, AuNPs can be conjugated with several functionalizing moieties such as ligands, therapeutic agents, peptides, proteins etc. In this study, we aimed to prepare cefotaxime-loaded gold nanoparticles (C-AuNPs) and evaluate their antibacterial efficacy against various bacterial strains. For this purpose, we challenged the efficiency of cefotaxime, itself, to act as a reducing and capping agent for the preparation of AuNPs and be loaded onto the surface of gold nanoparticles. Our results indicated the formation of C-AuNPs using a facile one-pot synthesis method, in which CTX efficiently acted as a reducing/capping agent (Figure 1). This method offered the advantage of permitting the synthesis of AuNPs and concomitant loading of the drug (CTX) from the same reaction mixture. Furthermore, this method nullified the use of an external chemical or biomolecule as reducing/capping agents and thus avoided the existence of residual contaminations that might interfere with the antibacterial results. Herein, the synthesis of the C-AuNPs was confirmed by a gradual color change of the reaction solution to ruby red from light yellow after incubation with CTX antibiotic. This color transformation is attributed to the surface plasmon resonance (SPR) that occurred in C-AuNPs [22,38]. 

### 3.2. Characterization of Cefotaxime-Loaded Gold Nanoparticles (C-AuNPs)

#### 3.2.1. UV–Visible Spectroscopy

**Figure 2:**
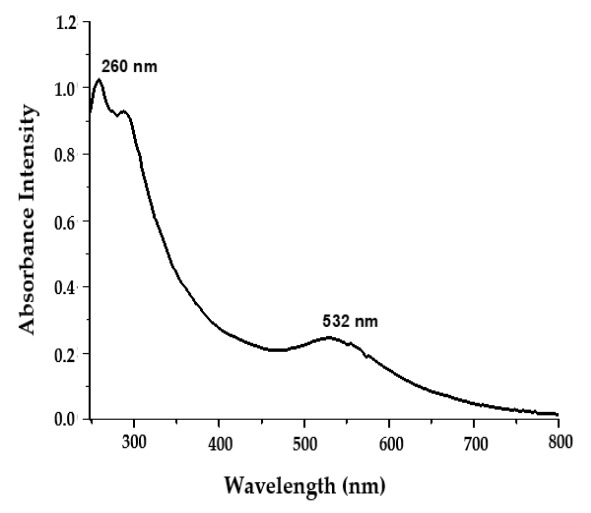
UV–Visible spectra of C-AuNPs showing a characteristic surface plasmon absorption band at 532 nm.

The surface plasmon resonance (SPR) of noble metal nanoparticles is a distinctive phenomenon ascribed to high electromagnetic fields around particle vicinity, which boosts all radiative characteristics such as absorption and scattering [35]. In the current study, UV–Visible spectroscopy was adopted to verify the synthesis of AuNPs. The existence of a surface plasmon absorption band at 532 nm, which corresponds to the plasmon band of AuNPs (Figure 2), confirmed the formation of AuNPs. Of interest, the appearance of an additional absorption peak at 260 nm (Figure 2), which is distinctive to CTX molecules [32], strongly affirmed the attachment of CTX molecules at the surface of the synthesized AuNPs. Our results demonstrated that CTX used in the reaction mixture efficiently acted as a potent reducing agent responsible for reducing gold salts to AuNPs. Furthermore, CTX helped stabilize the C-AuNPs formed by preventing the aggregation of the synthesized AuNPs.

#### 3.2.2. Transmission Electron Microscopy

**Figure 3:**
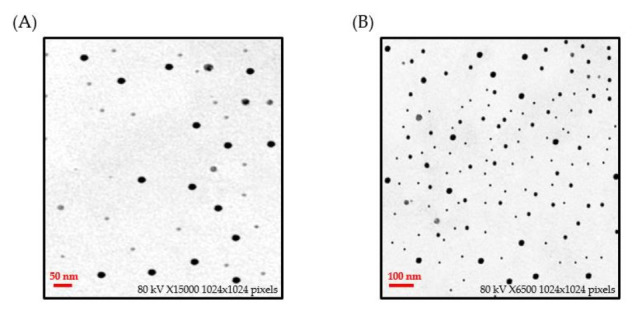
Transmission electron microscopy (TEM) micrograph images of C-AuNPs representing spherical monodispersed C-AuNPs with an average size of 21 nm at different magnification scales; (**A**) 15,000×, (**B**) 6500×.

The morphology, size, and shape of C-AuNPs were investigated using TEM analysis. AuNPs were rounded and evenly dispersed with no substantial aggregation, as depicted in TEM micrographs. The average size of C-AuNPs determined via TEM analysis was found to be 21 nm (Figure 3). The size calculation through TEM is based on the transmitted electrons, which provide information on the inorganic core only without including the information of the hydration layer.

#### 3.2.3. Size Determination via Dynamic Light Scattering

**Figure 4:**
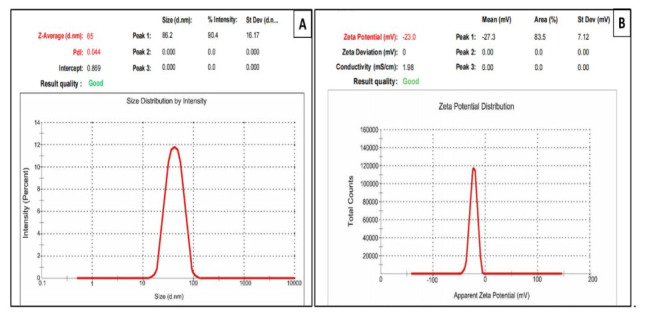
(**A**) Particle size analysis by dynamic light scattering (DLS, 65 nm), (**B**) Zeta-potential (−23 mV) of C-AuNPs.

The dynamic light-scattering (DLS) technique was also employed to estimate the particle size of the synthesized C-AuNPs. As depicted in Figure 4A, C-AuNPs showed an average particle size of 65 nm. The relatively higher particle size estimation by DLS, compared to that determined by TEM, might be attributed to DLS determining the size of nanoparticles in a hydrated state. When nanoparticles pass through a liquid medium, a thin electric dipole layer of the solvent shields their surfaces; consequently, DLS determines the size of not only nanoparticle’s inner inorganic core but the solvent sheath adhered to the nanoparticle core as well [22,39]. On the other hand, in TEM analysis, particle size estimation is undertaken in the dry state, and it reflects the exact diameter of particles excluding the effect of solvent sheath observed in DLS estimation [22].

#### 3.2.4. Zeta-Potential Study

Generally, the stability of colloidal systems is strongly influenced by the magnitude of the zeta potential. Particles with relatively high negative or positive zeta potential (>±20 mV) tend to repel each other and, consequently, there will be no tendency for particles aggregation [40]. Herein, the prepared C-AuNPs showed a zeta potential value of −23 mV, indicating good stability of the C-AuNPs (Figure 4B). The relatively high negative surface charge of C-AuNPs might be attributed, at least in part, to the presence of functional groups in CTX antibiotics, which further justifies the stability of the C-AuNPs.

#### 3.2.5. Fourier Transform Infrared Spectroscopy (FTIR)

**Figure 5:**
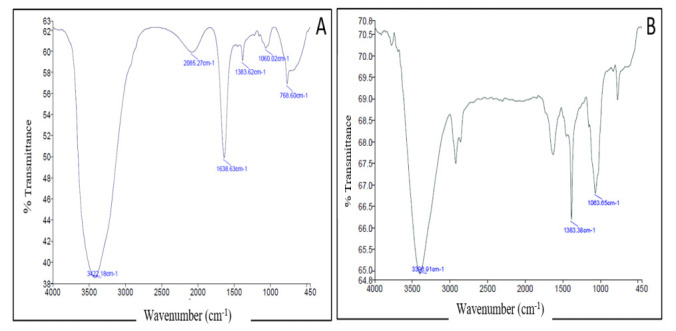
Fourier transform infrared (FTIR) spectra of (**A**) Pure CTX, (**B**) C-AuNPs.

FTIR spectroscopy was conducted to confirm the efficient loading of CTX onto the surface of AuNPs. The FTIR spectrum obtained from C-AuNPs was compared to that of pure CTX. The FTIR spectrum of pure CTX (Figure 5A) shows a characteristic absorption band at 3422.18 cm^−1^, corresponding to N–H and O–H groups (stretching vibrations). The additional peak at 1638 cm^−1^ represents amide N–H bending. The absorption band at 1638 cm^−1^ are designated for the C=C stretching and C=N stretching. The absorption band at 1060 cm^−1^ corresponds to C–O–C symmetric stretching. Of note, the obtained FTIR spectrum of C-AuNPs (Figure 5B) portrayed a significant reduction in N–H, C=N, C=C and C–O–C bands (Figure 5B), compared to the spectrum of pure CTX. These results might suggest the efficient capping/loading of CTX over AuNPs.

#### 3.2.6. Percent Loading of CTX over C-AuNPs

Loading efficiency represents one of the essential parameters for the characterization of nanoparticles. Herein, the loading efficiency of CTX onto AuNPs, manifested as the percentage of drug that is successfully attached/loaded onto the surface of the nanoparticles, was determined to be 83.94%. Out of 50 µg/mL of CTX incorporated in the reaction mixture, 41.97 µg/mL had been loaded onto the surface of AuNPs. This result indicates the efficient loading of CTX onto the surface of C–AuNPs and nullifies the significant loss of drug under our proposed preparation conditions.

### 3.3. Antibacterial Activity Analysis of C-AuNPs

The antibacterial abilities of C-AuNPs, pure CTX and AuNPs alone (as control) were validated by testing them against Gram-negative (*Escherichiacoli, Klebsiella oxytoca, Pseudomonas aeruginosa*) and Gram-positive (*Staphylococcus aureus*) bacterial strains. Following the experiment, it was noted that pure CTX and C-AuNPs diffused into the agar and strongly suppressed bacterial growth (as shown in Table 1). It is noteworthy that the concentration of CTX in C-AuNPs was only 4.19 µg/well in comparison to the concentration of pure CTX i.e., 20 µg/well. Thus, the data imply that using a very modest amount of C-AuNPs compared to pure CTX and AuNPs alone can be equally efficient against the tested bacterial strains. Our primary findings established that C-AuNPs outperformed CTX alone. This improved antibacterial potential of C-AuNPs over free CTX and AuNPs was possibly because AuNPs contain a significant pile of CTX, which is readily taken up by bacteria and escaped the degradation by bacterial enzymes. In addition, AuNPs itself are capable of causing bacterial DNA damage, presumably via direct interaction and blocking unwinding during the transcription process [41,42,43], that might have resulted in high antibacterial potential. The results are shown in Table 1 as a zone of inhibition.

### 3.4. Determination of Minimal Inhibitory Concentration of CTX and C-AuNPs

**Figure 6:**
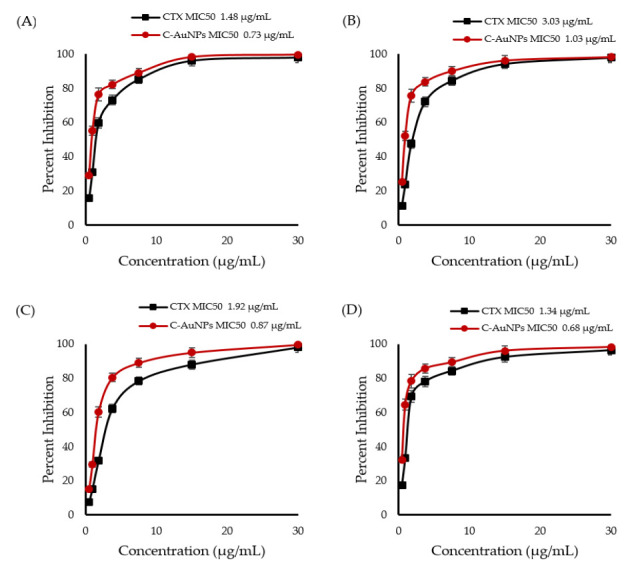
Minimum inhibitory concentration (MIC) of CTX and C-AuNPs against (**A**) *Escherichia coli*; (**B**) *Klebsiella oxytoca*; (**C**) *Pseudomonas aeruginosa. (***D***) Staphylococcus aureus.* The data represent the means ± standard errors of three independent experiments.

CTX and C-AuNPs MIC_50_ values represent the concentrations that inhibit 50% of the population of tested bacterial strains. The quantified MIC_50_ values were 1.48 µg/mL (CTX) and 0.73 µg/mL (C-AuNPs) for *Escherichia coli*; 3.03 µg/mL (CTX) and 1.03 µg/mL (C-AuNPs) for *Klebsiella oxytoca;* 1.92 g/mL (CTX) and 0.87 µg/mL (C-AuNPs) for *Pseudomonas aeruginosa;* and 1.34 µg/mL (CTX) and 0.68 µg/mL (C-AuNPs) for *Staphylococcus aureus,* respectively, as represented in Figure 6A–D.

Our MIC data demonstrated that C-AuNPs were significantly more effective at lower amounts than pure CTX and AuNPs against the aforementioned bacterial species. The attachment of CTX with AuNPs resulted in a reduced dosage of the CTX antibiotic, which could help in minimization of the antibiotic’s undesirable effects. The combined antibacterial properties of CTX and AuNPs might have accounted for the C-AuNPs’ increased antibacterial efficiency. AuNPs not only served as a carrier for the antibiotic CTX but exhibited potential antibacterial effects by collapsing the membrane potential and shifting the bacterial cell wall’s ATP level [41]. However, CTX delivered by the C-AuNPs was responsible for blocking transpeptidation in the bacterial cell wall, preventing the peptidoglycan cell wall from being synthesized [44].

### 3.5. Hypothesis on Mechanistic Aspects of the Antibacterial Potential of C-AuNPs

Based on the results of the study performed, we propose the hypothesis that there is an effective AuNPs-mediated delivery of the cefotaxime sodium to the bacterial cell, as an ample amount (83.94%) of CTX was attached onto it. The overall antibacterial effect of C-AuNPs might have resulted from the increased percentage of CTX molecules per unit volume of the system. CTX loaded on AuNPs might have gained easy entry into the Gram-positive bacterial cell due to increased porosity in the cell wall [45]. In addition, it has been observed that AuNPs could interact with lipopolysaccharide and protein present on the outer membrane of Gram-negative bacterial strains [41,43,46]. This might have aided the infiltration of AuNPs to deliver CTX effectively in the Gram-negative strains. Furthermore, modified architecture (the conjugation form of CTX-AuNPs) might have resisted active efflux pump as well [21]. Our hypothesis on AuNPs-loaded antibiotic is supported by the findings of Rai et al. [10], Shaikh et al. [23] and Alshammari et al. [26].

## 4. Conclusions

In this study, we successfully introduced a novel one-pot synthesis method of cefotaxime-loaded gold nanoparticles (C-AuNPs), in which cefotaxime itself acted as a reducing and capping agent. The synthesized C-AuNPs were highly stable (ζ potential ~ −23 mV) with an ample amount of CTX (loading efficiency 83.94%) loaded onto it. Most importantly, the antibacterial analysis demonstrated that the efficacy of CTX is substantially increased upon loading onto the surface of AuNPs; where much lower concentrations of C-AuNPs could inhibit the growth of tested Gram-negative and Gram-positive bacterial strains as compared to free CTX. To sum up, loading CTX onto metallic nanoparticles like gold might reduce the CTX treatment dosage with increased potency. Nonetheless, further investigation is required to evaluate C-AuNPs in vivo activity and/or safety before being considered as suitable drug carriers in the medical field.

## Figures and Tables

**Table 1:** Standard drug analysis for Gram-positive and Gram-negative bacterial strains.

Zone of Inhibition (mm)				
Sample	*Escherichia coli*	*Klebsiella oxytoca*	*Pseudomonas aeruginosa*	*Staphylococcus aureus*
CTX (20 µg/well)	28 ± 0.5 mm	17 ± 1.2 mm	21 ± 1.5 mm	15 ± 0.8 mm
C-AuNPs (4.19 µg/well)	26 ± 0.7 mm	15 ± 0.9 mm	20 ± 0.8 mm	13 ± 1.0 mm

Data are represented as mean ± standard deviation of three independent experiments performed under identical experimental conditions.

## Data Availability

Not applicable.
